# Love is Elsewhere: Internal Migration and Marriage Prospects in China

**DOI:** 10.1007/s10680-023-09658-3

**Published:** 2023-03-02

**Authors:** Wanru Xiong

**Affiliations:** The Hong Kong University of Science and Technology (Guangzhou), Urban Governance and Design Thrust, Guangzhou, China

**Keywords:** Internal migration, Marriage market, Assortative matching, China

## Abstract

Migration and marriage are major life events that might interact and be jointly decided. Places with good labor market opportunities may or may not provide good marriage options. In this paper, I quantify gains and losses in marriage prospects for unmarried migrants and natives during the population redistribution driven by internal migration. I also examine how the experiences differ by individual characteristics and regional factors. The analysis measures marriage prospects using the availability ratio (AR) with adaptive assortative matching norms for every unmarried individual from sample data of the 2010 China population census. The AR quantifies the intensity of competition for suitable partners in the local marriage market. I compare (1) migrants’ current AR with an alternative AR if the migrant returned to the hometown and (2) natives’ AR with a hypothetical AR if all migrants returned to their hometown. The first comparison shows that among migrants moving for labor market opportunities, most women have higher ARs (better marriage prospects) in the place of residence than in their hometown, especially those of rural origin. In contrast, migrant men’s ARs mostly decrease after migration except for the best educated. The second comparison reveals small negative externalities of internal migration on ARs for native women but positive impacts for some native men. The results suggest a conflict between labor market opportunities that dominate internal migration decisions and marriage market opportunities in China. This study demonstrates a method to quantify and compare marriage prospects and extends the literature on how migration and marriage interact.

**Supplementary Information:**

The online version contains supplementary material available at 10.1007/s10680-023-09658-3.

## Introduction

People migrate for a degree, a job, a significant other, or in general a better future. Sometimes these ends point toward one direction but sometimes not. When they are not, people face trade-offs. One dilemma that is common for young adults may be between labor market opportunities and marriage market options (Grossbard-Shechtman, 1984; Oppenheimer, 1988). Migration that facilitates a match in the labor market might influence the prospects available in the marriage market and vice versa (Stark, 1988). The migration decision thus involves a comparison of potential outcomes in both markets between the origin and the destination (De Jong, 2000). In the labor market, the difference is visible in terms of job opportunities and wages. In the marriage market, the expected prospect is a function of the number and availability of suitable partners, local matching norms, the relative position of the agent in the local marriage market, and luck (Becker, 1973; Choo & Siow, 2006; Lewis & Oppenheimer, 2000; South & Lloyd, 1992b). Independent individuals evaluate their options under constraints and make a move or stay. Families make a collective migration decision to maximize the total welfare (Blau et al., 2000; Chiappori et al., 2018; Clark & Lisowski, 2019). These actions aggregate to generate a population spatial redistribution that affects the equilibrium in both markets (Stark, 1988). Migrant workers may change the sex ratio in the local marriage market, while incoming brides and grooms increase the labor supply. These changes also affect natives in both the origin and the destination places, generating externalities of migration (Betz & Simpson, 2013; Borjas, 2006; Tabellini, 2020). The linkages between migration and marriage, between the labor market and the marriage market, and between migrants and natives, constitute a dynamic system in which agents’ decisions are interdependent.

Researchers have long studied the relationship between migration and marriage and the joint decision of these two major life events (Kulu & Milewski, 2007; Mincer, 1978; Mulder & Wagner, 1993; Rossi, 1955; Stark, 1988; Yeung & Mu, 2020). Marriage and cohabitation are important considerations promoting or deferring migration (Flowerdew & Al-Hamad, 2004). Moving for marriage is especially common for women where patrilocal marriage prevails (Davin, 2005, 2007; Fan & Huang, 1998; Kaur, 2004). Marriage opportunities in wealthier places attract migrants both within and across the national border (Chong, 2014; Constable, 2005; Ishii, 2016; Lin & Ma, 2008). Relationship dissolution also motivates relocation (Cooke et al., 2016; Feijten & Van Ham, 2007; Ferrari et al., 2019; Flowerdew & Al-Hamad, 2004; Mikolai & Kulu, 2018; Wall & Von Reichert, 2013). On the other hand, migration separating couples might affect marital quality and stability, but the direction of the effects depends on the purpose and duration of migration (Bhargava & Tan, 2018; Compernolle, 2021; Frank & Wildsmith, 2005; Li, 2018; Mu & Yeung, 2020; Muszynska & Kulu, 2007).

Less is known about the effect of migration on the marriage prospects of the unmarried (Yeung & Mu, 2020). One line of such literature focused on assortative matching in the receiving community, showing administrative constraints and social or cultural barriers against intermarriage between migrants and natives (Mu & Yeung, 2020; Qian & Qian, 2020; Tian & Qian, 2021). However, unmarried migrants might have more options after moving to a populous and active marriage market, so it is uncertain whether their marriage prospects improve or deteriorate. Few studies addressed this question in the context of internal migration, for which ethnic segregation is less of a problem than with international migration. One study examined how gender-selective internal migration affects regional mating chances in Germany (Eckhard & Stauder, 2018). There is also a lack of attention on how internal migration affects the marriage prospects of natives. The gaps exist for several reasons. First, it is difficult to measure marriage prospects of the unmarried because the prospects depend on the individual relative situation in a local context and have few behavioral indicators (Eckhard & Stauder, 2019). Second, there is an additional challenge in measuring the unrealized potential outcome, namely the marriage prospects for migrants in their hometowns and for natives under the hypothetical scenario of no migration (Eckhard & Stauder, 2018). Third, the association between migration and follow-up changes in marital status may not guarantee a causal interpretation due to unobserved confounders and reverse causality (Mulder & Wagner, 1993).

This study focuses on how internal migration affects marriage prospects of unmarried migrants and natives in China. The research questions are as follows: Do unmarried migrants moving for labor market opportunities improve their marriage prospects after migration? Do unmarried natives benefit from the population spatial redistribution? How do these gains and losses vary by sex, education, and residency status? Are socioeconomic factors of the receiving place predictors of these changes?

In this study, I review patterns of internal migration and assortative matching in China and propose several hypotheses about changes in marriage prospects for the unmarried due to internal migration. The hypotheses concern differential experiences by sex, migration status, and socioeconomic status. To test the hypotheses, I quantify individual marriage prospects in the local marriage market in 2010 using an Availability Ratio (AR) with adaptive local assortative matching norms regarding age, education, and residency status. The AR essentially quantifies the intensity of competition for suitable partners, and a higher AR means a more favorable situation (Goldman et al., 1984). I compare (1) migrants’ AR in their current place of residence with an alternative AR if the migrant returned to the hometown and (2) natives’ AR with a hypothetical AR if all migrants returned to their hometown. I also do regression analyses to estimate and compare conditional group means of ARs by sex, education, residency status, migration status, and regional factors. The results reveal significant sex differences in changes in ARs due to internal migration. The first comparison shows that among migrants moving for reasons related to the labor market, most unmarried women have higher ARs (better marriage prospects) in the place of residence than in their hometown, especially those of rural origin. In contrast, migrant men’s ARs tend to be lower after migration except for the best educated. The second comparison reveals that native women mostly suffer from small negative impacts of the population redistribution, whereas native men without college education mostly benefit from it. On average, migrant women have better marriage prospects than native women, but migrant men have worse marriage prospects than their native counterparts. Regional factors are not strong predictors of the AR, except that a higher urbanization rate is associated with better marriage prospects for men and a higher percentage of tertiary industry in GDP is positively associated with marriage prospects for women. These findings, being largely consistent with the hypotheses, reveal a conflict in the spatial distribution of labor market opportunities and marriage market options in China.

## Background

### Internal Migration in China

Mass voluntary internal migration in China began in the early 1990s, moving mainly from rural to urban areas and from inland provinces to coastal cities (Chan & Zhang, 1999). Before that, the household registration system (“hukou” system) embedded in the planned economy restricted internal migration. The system registered every citizen’s unique household address and related dichotomized agriculture (rural) or non-agriculture (urban) residency status (referred to as “hukou type”). The record might update upon college enrollment, job transfers, marriage, and divorce. The system continues to function but no longer as a restriction on relocation. Migrants can move to, stay in, and work in a new place without being able to change their hukou. Without a local hukou, they and their children do not have equal access as natives with local hukou to public school, social security, certain job positions, and they might need to earn additional qualifications to own a car and an apartment in the receiving place. These benefits attached to hukou differ by location and more significantly by urban/rural hukou type (Chan, 2019; Cheng & Selden, 1994; Song, 2014; Zhang et al., 2019). Nonetheless, internal migration increased in size rapidly since the early 1990s, growing from 21 million in 1990 to 221 million in 2010. Internal migration here counts domestic migrants, who reside in a prefecture (an administrative unit in China, explained in Sect. 3.2.1) other than the registered household address.

In 2010, the year of focus in this study, domestic migrants accounted for 16.2% of the total population in China. Among them, 80% had rural hukou type and around one-third moved across province (National Bureau of Statistics [NBS], 2012). The top receiving provinces were Guangdong, Zhejiang, Shanghai, Jiangsu, and Beijing, which were more developed provinces around coastal areas. Migrants mainly came from the neighboring inland provinces, such as Anhui, Sichuan, Henan, Hunan, and Hubei (Qiao & Huang, 2013). Provincial data show a strong positive association between the average disposable income in urban areas and the inflow migration rate, and a negative association between the average disposable income in rural areas and the outflow migration rate, suggesting that labor market opportunities are strong driving forces of internal migration (Qiao & Huang, 2013). Disparities in labor market opportunities and gains also explain the flow from rural to urban areas (Chan, 2013). Migration can be short term or long term. Some migrants settle in the destination and only return home temporarily during the Spring Festival. Some migrants move back for marriage, childbearing, family, or health reasons (National Health & Family Planning Commission, 2017).

There are sex differences in internal migration. According to the 2010 population census, 46.2% of working-age domestic migrants (ages 16–59) were women. Among these migrant women, 68% migrated for work, 10.4% for education or vocational training, and 15.2% for marriage or with family. The respective percentages among migrant men were 83.4%, 9.4%, and 3.2%, showing that more men migrated for economic opportunities and more women migrated for family ties (NBS, 2012). The pattern is consistent with the patrilocal tradition that the bride moves to her husband’s home after marriage (Davin, 1998). Regarding marital status, 38.5% of migrant men and 33.4% of migrant women had never married, and 60.6% of migrant men and 65.2% of migrant women were married. Unmarried migrant women were more likely than men to settle in the receiving place by marrying a local resident (Davin, 2005, 2007; Fan & Huang, 1998; Xiang, 2015), whereas more migrant men returned to their hometown for marriage and long-term residence after accumulating enough wealth as a migrant (Mu & Yeung, 2020). Another difference is that the proportion with primary school or below education was larger among migrant women (17.2%) than men (11.2%), but the proportions with a college degree were very similar with the percentage being 18.2% for women and 18.6% for men (NBS, 2012). Sex differences in the volume, direction, and educational composition of internal migration may significantly reshape the spatial distribution of the marriageable population (He & Gober, 2003). Furthermore, migrant women, especially those from rural areas, gain economic independence and obtain personal autonomy through migration, which enable them to have more options in their marital decisions (Davin, 1998). On the other side, women who stay in or return to rural areas are often tied to the village after marriage to take care of the family and the farmland when their husbands pursue migrant work (Fan, 2003).

### Assortative Matching

Marriage is nearly universal in China (Ji & Yeung, 2014; Jones & Yeung, 2014). Getting married is regarded as the default life choice, and a good marriage is a symbol of social success (Yeung & Hu, 2016). Prevailing social norms for assortative matching are homogamy or female status hypergamy, meaning that women marry men of equal or higher socioeconomic status than themselves (Han, 2010; Qian, 2012; Xu et al., 2014). These are a continuation of preferences in traditional arranged marriages that favor a husband with equal or higher education, income, and social status, and a younger wife with a gentle nature and attractive appearance (Xia et al., 2013). These preferences remain strong despite the shift from arranged to love-based mate selection, partly because men’s economic resources are supposed to support the high cost of raising children and rising consumption aspiration (Raymo et al., 2015). Such a prevailing expectation of gender-specific family obligations and the presumption of marriage as a prerequisite for childbearing reflect persistent patriarchal gender ideologies and gender inequalities both within and outside the family (Bumpass et al., 2009; Yeung & Hu, 2013; Yu & Xie, 2015).

Realization of assortative matching preferences is constrained by opportunities to meet a suitable partner, which depends on many factors, such as the size and composition of the local marriage market, third-party control, and geographical as well as social segregation (Kalmijn, 1991, 1994, 1998; Lewis & Oppenheimer, 2000). Research in Western countries has found that small ethnic/racial or religious group sizes (Blau et al., 1982; Kalmijn, 1991; Kalmijn & Van Tubergen, 2010), imbalanced sex ratios (Abramitzky et al., 2011; Lichter et al., 1992; Qian & Preston, 1993; Schoen, 1983), educationally or geographically sparse market (Blau & Schwartz, 2018; Lewis & Oppenheimer, 2000) are barriers against desired assortative matching. In China, the corresponding factors of marriage opportunities are spatial concentration of the marriageable population, urban and rural segregation, imbalanced sex ratios, and population composition by age, education, and socioeconomic status. These structural arrangements set the context of assortative matching in the local marriage market, in which the ones in a favorable position have higher probabilities and greater bargaining power to realize their preferences (Kalmijn, 1998; Lichter et al., 1991). Those in a less favorable position might extend their search, pursue higher socioeconomic status, or adapt their preferences (Kalmijn, 1998; Lichter et al., 1995; Mu & Xie, 2014; Oppenheimer, 1988). When preferences for status homogamy or female hypergamy remain strong in the backdrop of increasing women’s educational attainment and economic independence, as observed in many East Asian societies, a mismatch between preferences and opportunities in the marriage market makes it numerically difficult for some groups to get married (Raymo & Iwasawa, 2005). In this case, opportunities to meet a suitable partner, or essentially the structure of the local marriage market, become the prime factor of assortative matching. This factor can be changed to some degree through relocation for a migrant and through the redistribution of the marriageable population for a native resident, which is a key point that the study aims to demonstrate.

### Hypotheses

Sex differences in internal migration and the social norms of assortative matching imply potential friction between matching in the labor market and in the marriage market. In this section, I propose four hypotheses about changes in marriage prospects of the unmarried due to internal migration based on these implications.

The first hypothesis is about different experiences by sex among unmarried migrants moving for reasons related to the labor market. Efficient matching in the labor market hinges on the alignment of the demographic composition of the labor force and the occupational structure in the local economy (Burdett & Vishwanath, 1988; Coleman, 1991). When migration is primarily motivated by labor market opportunities, the resulting population distribution might not be optimal for the marriage market, in which matching follows different rules (Chiappori, 2020; Choo & Siow, 2006; Lichter et al., 1995). One implication is that gender-segregated occupations might give rise to an imbalance in the marriage market (He & Gober, 2003).

For example, in top-tier cities where high-paying jobs concentrate, aspiring young migrants from all over the country make the most out of their skills and labor (Fu & Gabriel, 2012; He & Wu, 2017; Liu & Shen, 2017; Liu et al., 2015; Zhou et al., 2018). Some of these jobs disproportionately absorb men, such as technician, engineer, and programmer (NBS, 2012). In smaller cities and towns, the more desirable jobs are in the public sector, such as civil servant, teacher, and doctor. These stable and decent jobs are good options for women looking for work-family balance (Yang, 2013). Therefore, big cities tend to be good labor markets for young men and women but favorable marriage markets only for women and some men with outstanding traits (Edlund, 2005). Small cities and towns provide plenty of decent jobs for women but fewer for men, leaving women with a limited pool of marriageable men (Ouyang & Ma, 2019). That means migrant men and native women are more likely to face a conflict between opportunities in the labor market and the marriage market.

Furthermore, when making the migration decision, single men might prioritize labor market opportunities and temporarily sacrifice marriage prospects, as success in the labor market boosts their chance of marriage (Mu & Xie, 2014; Qian & Qian, 2014; Yu & Xie, 2015). Unmarried women might find marriage prospects equally important as job opportunities, partly because both could be potential ways for upward mobility (Chen, 2018; Gaetano, 2017; Xu et al., 2000). However, neither way is easy. In the marriage market, women feel time pressure due to the “gendered double standard of aging” wherein women are devalued as they age (England & McClintock, 2009). In the labor market, women face the gender wage gap, discrimination, and social-cultural obstacles to geographical mobility (He & Gober, 2003; Kuhn & Shen, 2013; Thadani & Todaro, 2019). Therefore, unmarried women need to balance time-constrained marriage prospects and restricted labor market opportunities in their migration decision. As a result, we expect to see that among those who decide and manage to move, single women are more likely to have improved marriage prospects than men.

#### Hypothesis 1

Migrant women are more likely to improve their marriage prospects via migration than migrant men.

The second and third hypotheses focus on variations across socioeconomic groups among migrants. For women, paths to success in the labor market and the marriage market can be diverging. Women with higher socioeconomic status have a lower chance of achieving status hypergamy because fewer men meet their high standards (Ji, 2015; Qian & Qian, 2014). Women with professional skills and a desire for career success might find marriage less necessary and desirable than women in pursuit of a traditional role in the family, so their migration decision might be driven by education or labor market opportunities that are highly concentrated in top-tier cities (Gaetano, 2014; Ji, 2015; To, 2015). Women without specialized skills feel less restricted by the location of the institution or the industry, and therefore can move to improve their marriage prospects (Davin, 2005; Fan & Huang, 1998). The room for improvement can be considerable given the regional and rural–urban differentials in education and socioeconomic development.

#### Hypothesis 2

Women with lower socioeconomic status gain more in marriage prospects via migration than those with higher socioeconomic status.

For men, marriage prospects are closely tied to socioeconomic status, so the majority of migrant men prioritize labor market opportunities (NBS, 2012). In doing so, those with better traits are more likely to succeed in the marriage market competition and match with native or migrant women with a similar or inferior background. Migrant men with a less desirable background might sacrifice their marriage prospects because their relative ranking in the marriage market is often lower in the destination than in their hometown. Overall, men’s marriage prospects are less likely to be affected by relocation when they have better socioeconomic status.

#### Hypothesis 3

Men with lower socioeconomic status lose more in marriage prospects via migration than those with higher socioeconomic status.

The fourth hypothesis concerns natives. Although natives do not participate in internal migration, their marriage prospects might be affected because the geographical redistribution of the population brings in or takes away their potential marital partners and competitors (Eckard & Stauder, 2018). The impact on natives thus can be termed as an externality of internal migration, meaning that it is an indirect cost or benefit to an uninvolved third party arising from migrants’ activities (Mankiw, 2014). The externality can be positive when suitable marital partners move in or competitors move out, or negative when the two groups move in the other direction. A native’s experience might be inferred from the experience of a migrant of the same sex and traits, who is a competitor for the native in the local marriage market. In their hometown, when the migrant sacrifices marriage prospects and leave for better labor market opportunities, a native resident gains from having fewer competitors. However, the native’s gains might be discounted by the outflow of potential matches. In the receiving place where a migrant improves marriage prospects, a local resident of the same sex and traits faces additional competition. Again, such a loss in marriage prospects would be compensated by the inflow of potential matches. Therefore, we might observe that migrants and natives of the same sex and traits have different experiences during the internal migration process.

#### Hypothesis 4

Migrants and natives of the same sex and traits experience the opposite change in marriage prospects during the geographical redistribution of the marriageable population.

The following section describes empirical strategies to test these hypotheses.

## 3. Data and Methods

### Data and Sample

The individual-level sample data of the 2010 population census provide a unique opportunity to study individual relative positions in local marriage markets in China. The dataset is a 1% random sample of households that were randomly selected to complete the long-form survey in the population census (10% of total households). The long-form survey collected information about migration history and marital status, in addition to the basic demographic characteristics collected in the short form, including sex, age, education level, and household registration residency status (hukou type). The data contain information about both residency at the census time and the household registration address, so they can be used to study marriage prospects for migrants in the current place of residence and their hometown.

The analytical sample consists of 121,179 young adults aged 20–34 who were unmarried at the census time, including the never married (97.6%), divorced, and widowed. Restricting the sample to the never married makes little difference. The sample is representative of the population aged 20–34 in mainland China except in Xinjiang, Tibet, and Qinghai provinces, from which the observations are excluded due to very low population density (< 15 persons per square kilometer). In the analytical sample, 21.9% are migrants who moved across prefectures and had been away from their hukou address for over 6 months. Among them, 95.8% of men and 94.5% of women moved for reasons related to labor market opportunities, including for work, business, education, and vocational training. Others moved with family members, for marriage, or for other reasons. The percentage of migrants moving for labor market related reasons among the population aged 20–34 is much higher than that in the total population of migrants. Table 1 shows the descriptive statistics of the analytical sample.

**Table 1:** Descriptive statistics of the analytical sample (unmarried, ages 20–34)

Sex	Men		Women	
Migration status	Migrant	Native	Migrant	Native
*N*	15,484	53,828	11,057	40,810
Percentage among men/women	22.34%	77.66%	21.32%	78.68%
Mean age	23.36	23.95	22.78	23.29
*Percentage within group*	%	%	%	%
Marital status				
Never married	99.43	97.37	99.54	96.94
Divorced	0.54	2.43	0.43	2.49
Widowed	0.03	0.19	0.03	0.57
Hukou type				
Rural hukou	76.09	67.14	72.18	64.49
Urban hukou	23.91	32.86	27.82	35.51
Current residence				
Rural	10.79	50.31	7.94	46.30
Urban	89.21	49.69	92.06	53.70
Education level				
Primary school or below	2.77	7.70	1.59	5.70
Junior high school	38.02	42.75	32.39	38.96
Senior high school	21.99	21.94	21.82	21.45
College and above	37.22	27.60	44.20	33.90
Reason for migration				
Work/business/education/training	95.77	–	94.80	–
Marriage/family	2.66	–	3.30	–
Others	1.57	–	1.90	–

The calculation uses information from the same data about unmarried persons aged 14–41 who could be potential partners for those in the analytical sample. It also uses information about marriages formed after 2005 and lasted till the census time in 2010 as recorded in the census data (referred to as “the marriage sample”) to infer prevailing local assortative matching patterns and calculate weights. Same-sex marriage is not recognized in China, so this analysis focuses exclusively on opposite-sex marriage.

### Measure

#### Size of the Local Marriage Market

This study defines the local marriage market at the prefecture level. A prefecture in China, formally known as a prefectural-level division, is the second level of the administrative structure, ranking below a province and above a county. There are 333 prefectures in mainland China. According to the population census in 2010, the smallest 10% of the prefectures had on average 480 thousand people in each prefecture and the largest 10% of the prefectures had on average 10 million. Within a prefecture, social and economic connections construct a reasonably well-connected social network. Two individuals living in the same prefecture have a realistic chance to meet each other via daily contacts, work connections, family relationships, and friendship circles, which are the common ways of meeting a partner. Online-dating websites use the prefecture-level location in their matching algorithms so that matched users may develop their relationships offline. Nonetheless, defining the geographic scale of the local marriage market in a world with increasing connectivity is conceptually difficult and always requires some degree of subjectivity. The choice is also constrained by the granularity of the data in an empirical study. The other two options, namely province and county, are too large or too small, respectively, to define a local marriage market.

#### Local Assortative Matching Patterns

The analysis measures local assortative matching patterns at the province level, a level above the prefecture, using marriages formed after 2005 and lasted till the census time in 2010. Allowing assortative matching patterns to vary by region alleviates the endogeneity problem that matching patterns adapt to local marriage market opportunities. The decision to use the province instead of the prefecture as the unit is a compromise between using information about marriages from recent years and proximate geographical areas. Some prefectures do not have enough observations of recent marriages to infer robust matching patterns and variations in matching patterns across prefectures within a province tend to be small. For each of the 30 provinces, I calculate the empirical distribution of the age gap and the education gap between the husband and the wife. The distributions show similar curves with moderate variations across provinces (see Online Resource 1).

#### Availability Ratio

The analysis measures individual marriage prospects using an extended version of the Availability Ratio (AR) developed by Goldman et al. (1984), which quantifies the intensity of competition in the marriage market. The extension adds weights in the calculation of the AR. The weighted AR is the ratio of the weighted number of potentially suitable marital partners for a particular individual (referred to as “matches”) to the weighted average number of matches of this individual’s matches (referred to as “competitors”), with weights reflecting local matching patterns. In essence, it is the number of matches divided by the average number of competitors (Goldman et al., 1984). An AR greater than one indicates a favorable situation for the individual in the local marriage market, whereas an AR less than one indicates a difficult one. For example, if a woman has 100 matches and on average each of these suitable men has 100 matches, then the local marriage market is well balanced and the AR for this woman is $100/100=1$. If each of those suitable men has on average 200 matches, then the situation is difficult for this woman as reflected by an AR of $100/200=0.5$.

The weighted AR has some special features. First, matching is reciprocal, meaning that if a man is defined as a match for a woman, then the woman is also a match for the man. Second, the AR incorporates competition from other groups of the same sex, approximating various assortative matching preferences. For example, assuming that men prefer a woman of the same or younger age, then the AR for a 27-year-old woman has a 27-year-old man as one of the matches in the numerator, and the number of women aged 25 as part of its denominator because these women can be competitors, i.e., matches for the 27-year-old man. Therefore, the AR for a woman of certain traits would not be the reciprocal of the AR for a man of the same traits. Third, the AR uses weights to reflect the degree of preference for a certain match, allowing for the fact that a potential match with less favorable traits adds less to one’s marriage prospects. The weights are derived from the empirical distribution of local matching patterns as described in Sect. 3.2.2.

The AR is a more sophisticated measure of marriage prospects than population sex ratios (Eckhard & Stauder, 2019; Fossett & Kiecolt, 1991; Goldman et al., 1984; Lampard, 1993; South & Lloyd, 1992a, 1992b). It focuses on unmarried persons in a local marriage market at a given time, captures competition from other groups of the same sex, counts matches from cohorts of different sizes, incorporates assortative matching patterns by measurable factors, and takes into account empirical local mating patterns (Eckhard & Stauder, 2019; South & Llyod, 1992a, 1992b). An empirical test shows that AR is a better predictor of union formation than age-specific sex ratios (Eckhard & Stauder, 2019). The standard AR has been used to describe marriage opportunities in the USA (Bhrolchain & Sigle-Rushton, 2005; Goldman et al., 1984; South & Lloyd, 1992a, 1992b), Germany (Eckhard & Stauder, 2018, 2019), England and Wales (Bhrolchain, 2008), and India (Billig, 1991, 1992). The defining features of a match—the key specification in constructing the AR—used in these studies include age, race, caste, education, employment status, and geographic location.

In this analysis, a match is defined by basic features of a potential suitable partner, including age, education, prefecture of residence, and hukou type. As in previous studies, the definition does not consider other assortative matching factors such as income, wealth, family background, personality, and physical appearance, which are difficult to measure and unavailable in the census data.

A match is defined as follows: First, a match must be currently unmarried, including the never married, divorced, and widowed. For simplicity, the operational definition of a match does not differentiate between the never married and the divorced/widowed since the majority in the analytical sample has never married (97.6%).

Second, two persons need to reside in the same prefecture and have the same hukou type to become a matched pair. The hukou restriction depicts the urban–rural boundary in the marriage market (Qian & Qian, 2017; Wei & Zhang, 2016; Zhou, 2019). The assumption about rural–urban hukou segregation is supported by the fact that only 7.9% of the husband and the wife have different hukou types in the marriage sample. Two robustness checks relax this assumption (see Sect. 3.3).

Third, a match must be within a certain age range to account for age preferences in matching. In the marriage sample, each of the nine age gaps between – 2 to 6 (husband–wife) accounts for over 3% of the couples, and in total 87.8% have an age gap in this range, with a mean of 1.97. Therefore, I define a match to have an age gap between – 2 and 6 years and use the percentage of marriages with the age gap among marriages with an age gap between – 2 to 6 as the weight (see Online Resource 1). Those outside the range are counted as zero matches.

Fourth, a matched pair should have equal or neighboring education levels, assuming educational assortative matching. Education is classified into four levels: illiterate and primary school, junior high school, senior high school, and college and above. According to the marriage sample, over 95% of couples have equal education or a one-level gap in their education. Therefore, I define a pair with an education gap of no more than one as a matched pair. The weight is the scaled percentage of marriages between the person’s education and the potential match’s education among all marriages involving the person’s education (see Online Resource 1).

The final weight of a specific match is the product of the weights of age and education. It is assumed that the two weights are independent because the marriage sample size in each province is not large enough to infer a joint distribution. The scale of the weight does not affect the interpretation of AR as the weight appears on both the numerator and the denominator of the ratio. The calculation of the AR is performed in the following steps:

1. Let $M_{ijkl}$ be the number of unmarried men (${\text{Man}}_{ijkl}$) of age $i$ (single-year), education level $j \in \left\{ {1{ }\left( {{\text{illiterate and primary school}}} \right), \ldots ,{ }4{ }\left( {\text{college and above}} \right)} \right\}$, and hukou type $k$ in prefecture $l$; similarly, let $W_{{i{^{\prime}}j{^{\prime}}kl}}$ be the number of unmarried women (${\text{Woman}}_{{i{^{\prime}}j{^{\prime}}kl}}$) of age $i{^{\prime}}$, education level $j{^{\prime}}$, and hukou type $k$ in prefecture $l$.
2. The matching rule is represented by province-specific weights: let $\omega_{{ii^{\prime}jj^{\prime}l}}$ be the weight of a match between a ${\text{Man}}_{ijkl}$ and a ${\text{Woman}}_{{i{^{\prime}}j{^{\prime}}kl}}$ of the same hukou type $k$ in prefecture $l$.
  - For non-matched pairs, i.e., $i - i^{\prime} \notin \left\{ { - 2, \ldots ,6} \right\}$ or $j - j^{\prime} \notin \left\{ { - 1, 0, 1} \right\}$, $\omega_{{ii^{\prime}jj^{\prime}l}} = 0$.
  - For matched pairs, $\omega_{{ii^{\prime}jj^{\prime}l}}$ is the product of the scaled percentage of recent marriages with an age gap $i - i^{\prime}$ and the scaled percentage of recent marriages between a husband with education $j$ and a wife with education $j^{\prime}$ in the province where prefecture $l$ locates.
3. The weighted count of suitable women for ${\text{Man}}_{ijkl}$ is ${\text{SW}}_{ijkl} = \mathop \sum \limits_{i^{\prime}} \mathop \sum \limits_{j^{\prime}} \omega_{{ii^{\prime}jj^{\prime}l}} \times W_{i^{\prime}j^{\prime}kl}$. Similarly, the weighted count of suitable men for ${\text{Woman}}_{{i{^{\prime}}j{^{\prime}}kl}}$ is ${\text{SM}}_{i^{\prime}j^{\prime}k} = \mathop \sum \limits_{i} \mathop \sum \limits_{j} \omega_{{ii^{\prime}jj^{\prime}l}} \times M_{ijkl}$.
4. The AR for ${\text{Man}}_{ijkl}$ is
  $${\text{Availability}}\;{\text{ratio}} \left( {{\text{AR}}_{ijkl} } \right) = \frac{{{\text{SW}}_{ijkl} }}{{\left( {\mathop \sum \nolimits_{i^{\prime}} \mathop \sum \nolimits_{j^{\prime}} \omega_{{ii^{\prime}jj^{\prime}l}} \times W_{i^{\prime}j^{\prime}kl} \times {\text{SM}}_{i^{\prime}j^{\prime}kl} } \right)/{\text{SW}}_{ijkl} }},$$

and the AR for ${\text{Woman}}_{{i{^{\prime}}j{^{\prime}}kl}}$ is

$${\text{Availability}}\;{\text{ratio}} \left( {{\text{AR}}_{{i^{\prime}j^{\prime}kl}} } \right) = \frac{{{\text{SM}}_{i^{\prime}j^{\prime}kl} }}{{\left( {\mathop \sum \nolimits_{i} \mathop \sum \nolimits_{j} \omega_{{ii^{\prime}jj^{\prime}l}} \times M_{ijkl} \times {\text{SW}}_{ijkl} } \right)/{\text{SM}}_{i^{\prime}j^{\prime}kl} }}$$

### Scenarios for Comparison

For each person, I calculate an AR based on their current prefecture of residence and a hypothetical AR as the potential outcome for comparison. The hypothetical scenario differs by migration status.

For migrants whose prefecture of residence is different from the prefecture of their household registration address (usually the hometown), I assign the person to the hometown prefecture to calculate an alternative AR, keeping the person’s other traits and everyone else’s residence constant. I use weights that reflect the matching patterns in the hometown province for the hypothetical AR. This AR for migrants thus measures the marriage prospects the migrant would have if he/she returned to the hometown. Comparing this hypothetical AR and the migrant’s current AR after migration reveals changes in marriage prospects for migrants due to migration.

For natives who stay in the prefecture of their household registration address, I calculate a hypothetical AR under the scenario of no migration. I assign *all* migrants to their hometown prefecture and keep everything else constant in the calculation. Differences between the real AR and this hypothetical AR for natives thus reflect the impact of internal migration on their marriage prospects. This hypothetical AR also applies to migrants, but its interpretation is different from the previous one, in which only the migrant of interest moves back to the hometown. This strategy is similar in concept to the hypothetical “migration-adjusted” AR developed in Eckhard and Stauder (2018).

For robustness checks, I calculate these ARs under different scenarios characterized by alternative matching rules. In one hypothetical scenario, I extend the geographic boundary of local marriage markets to include matches from the same province. This is intended to reflect an enlarged pool made possible by online dating and fast transportation. In a second one, I remove the segregation by hukou type to allow intermarriages between individuals of urban and rural origin, as recent reforms of the hukou system have been integrating urban and rural hukou (Cui & Cohen, 2015; Lui, 2017). In a third one, I remove the segregation by hukou type but separate the local marriage market into an urban one and a rural one, emphasizing the geographical and socioeconomic segregation between urban and rural areas.

### Regression

The study uses regression analysis in addition to graphical representations to analyze the changes in ARs due to migration. The regression models estimate the means of ARs and changes in ARs by sex and group, conditional on other factors that might be relevant to the outcome. Groups are defined by education (${\text{educ}}$), hukou type (${\text{hukou}}$), and migration status (${\text{migrant}}$). The control variables (${\text{control}}$) include age (and its quadratic form) and current urban/rural residence, which can be different from the hukou type. The analysis also tries to identify predictors of the AR in terms of demographic or socioeconomic factors of the prefecture of residence in 2010. The candidate prefecture-level predictors are (1) urbanization rate defined by the percentage of urban residents in the total population; (2) percentage of the population aged above 15 with above high school education; (3) percentage illiterate of the population aged above 15; (4) annual average disposable income of urban employee (log form); (5) average housing price (yuan/square meter, log form); (6) gross domestic product (GDP) per capita (log form); (7) percentage of GDP corresponding to secondary industry; (8) percentage of GDP corresponding to tertiary industry; (9) the number of enrolled college students (log form).

I estimate four sets of ordinary least square regression models. The first model concerns group means of changes in $\mathrm{ln}(\mathrm{AR})$ ($\mathrm{\Delta ln}(\mathrm{AR}))$ for migrants, where $\mathrm{\Delta ln}(\mathrm{AR})$ is the natural log of AR in the current prefecture of residence minus the natural log of the alternative AR if the migrant returned to the hometown. $\mathrm{\Delta ln}(\mathrm{AR})$ equals the natural log of the ratio of these two ARs. The model includes full interactions between sex, hukou type, and education level:

$$\Delta \ln ({\text{AR}}_{ij} ) = \alpha + \gamma \times {\text{sex}}_{i} \times {\text{hukou}}_{i} \times {\text{educ}}_{i} + \rho \times {\text{control}}_{i} + \varepsilon_{i} ;$$

The second one concerns group means of changes in $\ln \left( {{\text{AR}}^{\prime } } \right)$($\Delta \ln \left( {{\text{AR}}^{\prime } } \right)$) for natives, where $\Delta \ln \left( {{\text{AR}}^{\prime } } \right)$ is the natural log of the actual AR minus the natural log of the hypothetical AR under the scenario of no migration. Again, $\Delta \ln \left( {{\text{AR}}^{\prime } } \right)$ can be understood as the natural log of the ratio of these two ARs:

$$\Delta \ln ({\text{AR}}^{\prime}_{ij} ) = \alpha + \gamma \times {\text{sex}}_{i} \times {\text{hukou}}_{i} \times {\text{educ}}_{i} + \rho \times {\text{control}}_{i} + \varepsilon_{i} ;$$

The third model estimates the group means for the AR of all current residents by sex, controlling for age and urban/rural residence.

$${\text{AR}}_{ij} = \alpha + \beta \times {\text{sex}}_{i} \times {\text{migrant}}_{i} \times {\text{hukou}}_{i} \times {\text{educ}}_{i} + \rho \times {\text{control}}_{i} + \varepsilon_{i};$$

The fourth model tries to identify regional predictors of AR. The model uses cluster-robust standard errors to account for the correlation between prefectures within a province:

$$\ln ({\text{AR}}_{ij} ) = \alpha + \beta \times {\text{sex}}_{i} \times {\text{migrant}}_{i} \times {\text{hukou}}_{i} \times {\text{educ}}_{i} + \rho \times {\text{control}}_{i} + \theta \times {\text{predictors}} + \varepsilon_{i} .$$

### Limitations

The data and methods used in this analysis have several limitations. First, the analytical sample is a random sample of the total population aged 20–34, but the calculation of AR neither accounts for variations in sampling nor quantifies uncertainties due to technical difficulties. Second, the definition of migrants is based on the current residence and the hukou address and therefore excludes those who convert their hukou after migration. Third, local matching patterns are treated as exogenous, but they might be endogenous to the availability of suitable marital partners in the local marriage market (Mu & Xie, 2014). Fourth, the geographic boundaries of the local marriage market are defined at the prefecture level, but they are likely to be flexible in real life and to extend beyond the administrative boundaries. Fifth, the analysis uses education and hukou type as proxies for socioeconomic status, which might not capture the key factors in the marriage market. While these assumptions are necessary compromises due to data limitations, we need to be careful in interpreting the results.

## Results

### Changes in Marriage Prospects for Migrants

**Fig. 1:**
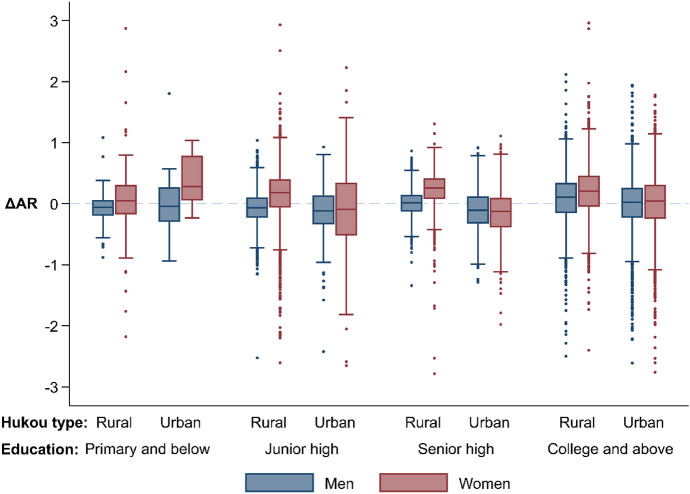
Boxplots of differences between the AR in the current place of residence and the AR in the hometown (ΔAR) for migrants moving for reasons related to the labor market by sex, education, and hukou type. *Note:* ΔAR = AR in the current place of residence − AR in the hukou address. This graph shows the values for migrants moving for reasons related to the labor market, such as work, business, education, and vocational training. *Source*: Sample data of 2010 China population census, author’s calculations

Figure 1 presents box plots (box-and-whisker plots) of the $\Delta {\text{AR}}$ by sex, hukou type, and education, highlighting the contrast by sex. The $\Delta {\text{AR}}$—the difference between the AR in the current prefecture of residence and the AR if the migrant returned to the hometown—quantifies changes in marriage prospects for migrants after migration. Figure 1 shows $\Delta {\text{AR}}$ s for the 95.3% of migrants moving for reasons related to the labor market. The corresponding figure for all migrants looks very similar (see Online Resource Figure A1). For each group, the box in the middle shows the interquartile range (IQR) with its upper bound (75th percentile) and its lower bound (25th percentile). The line subdividing the box represents the median value (50th percentile). The whiskers extend to include all data points within 1.5 IQR of the near quartile and stop at the largest/smallest such value. The dots beyond the whiskers mark the outliers (Cox, 2009). Few extreme values outside the range of the plot are not shown. A gray-dashed line representing zero difference separates the groups with improved marriage prospects above the line and the groups with a lower AR after migration below the line. Table 2 shows the estimated group means of $\Delta \ln \left( {AR} \right)$ controlling for age and current urban/rural residence in the same population. Figure 1 and Table 2 present the same comparison using different statistics: Fig. 1 uses the difference whereas Table 2 uses the ratio.

**Table 2:** Regression estimated conditional group means of differences between the natural log of AR in the current place of residence and the natural log of AR in the hometown ($\Delta {\text{ln}}\left( {{\text{AR}}} \right) = {\text{ln}}({\text{AR}}_{{{\text{residence}}}} /{\text{AR}}_{{{\text{hometown}}}}$) for migrants moving for reasons related to the labor market

Sex	Hukou type	Primary school or below	Junior high school	Senior high school	College and above
Men	Rural	− 0.223***	− 0.087***	0.014	0.120***
		(0.027)	(0.012)	(0.014)	(0.014)
Men	Urban	− 0.072	− 0.232***	− 0.174***	0.012
		(0.123)	(0.030)	(0.024)	(0.015)
Women	Rural	0.066	0.137***	0.326***	0.303***
		(0.043)	(0.014)	(0.016)	(0.014)
Women	Urban	0.346	− 0.139***	− 0.180***	0.036*
		(0.201)	(0.039)	(0.027)	(0.015)

Standard errors in parentheses. * *p* < 0.05, ** *p* < 0.01, and *** *p* < 0.001

A contrast between migrant men and women is evident in Fig. 1 and Table 2. For migrant women, the median of $\Delta {\text{AR}}$ s (Fig. 1) and the mean $\Delta \ln \left( {{\text{AR}}} \right)$ s (Table 2) are above zero (ratio > 1) except for those with urban hukou and junior/senior high school education (5.9% of migrant women), meaning that most of them have a higher AR in their current place of residence than in their hometown. Migrant women with rural hukou increase their ARs via migration more than their peers with urban hukou except for the least educated. Among migrant women with rural hukou, the better educated have a larger increase in the AR, whereas among those with urban hukou, the least educated gain the most. The plots and group means look different for migrant men. The median of $\mathrm{\Delta AR}$ s and the mean $\mathrm{\Delta ln}(\mathrm{AR})$ s are below zero (ratio < 1) for migrant men without a college degree. Within this group, those with rural hukou lose less than those with urban hukou except for the least educated. In contrast, the college-educated migrant men mostly have an increased AR in their current place of residence. Overall, 67.1% of migrant women and 48.3% of migrant men moving for reasons related to the labor market have a higher AR in their current place of residence than in their hometown.

The findings largely support Hypothesis 1 that migrant women are more likely than men to improve their marriage prospects via migration. The findings also give some evidence for Hypothesis 2 that migrant women with rural hukou gain more than those with urban hukou, and among migrant women with urban hukou, the less educated gain more. The contrast that migrant men with the best education have improved marriage prospects after migration while others lose supports Hypothesis 3. Robustness checks that compare the ARs using different matching rules give largely consistent results (see Online Resource Figures A2, A3, and A4). The patterns are even more evident under the scenario that allows for intermarriages between urban and rural hukou (Figure A3) and the scenario in which the local marriage market is segregated by current urban/rural residence instead of the hukou type (Figure A4).

Another comparison for migrants is between the current AR and the hypothetical AR assuming no migration (denoting as $\Delta {\text{AR}}^{\prime }$). Figure A5 (Online Resource) shows the box plots of $\Delta {\text{AR}}^{\prime }$ for migrants. The patterns are similar to those in Fig. 1, that in general migrant women benefit from the population redistribution caused by internal migration, while migrant men suffer. However, the gains and losses for migrants tend to be smaller in magnitude under the hypothetical scenario in which everyone stays in their household registration address (Figure A1) than under the more realistic scenario that only the migrant of interest returns to the hometown (Fig. 1).

### Changes in Marriage Prospects for Natives

**Fig. 2:**
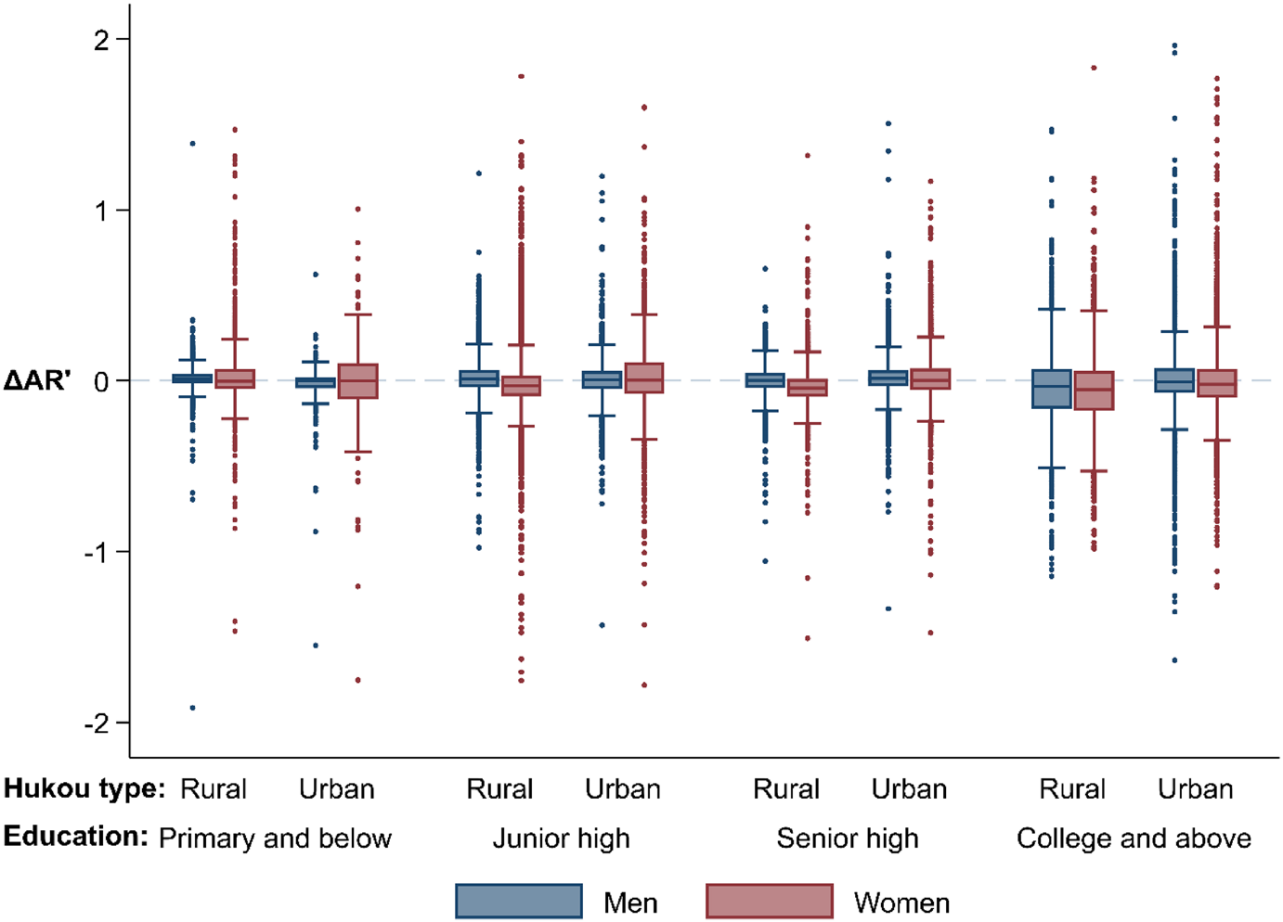
Boxplots of differences between the current AR and the AR assuming no migration for natives (ΔAR’) by sex, education, and hukou type. *Note:* ΔAR’ = AR in the current place of residence − AR assuming all migrants returned to their hukou address. *Source*: Sample data of 2010 China population census, author’s calculations

Figure 2 and Table 3 show changes in ${\text{AR}}^{\prime}$ s for natives, which quantifies the externality of internal migration on natives’ marriage prospects. Hypothesis 4 states that the experience of natives would be the opposite of the experience of their migrant counterparts, which can be tested by comparing Fig. 1 with Fig. 2 and comparing Table 2 with Table 3.

**Table 3:** Regression estimated conditional group means of differences between the natural log of the current AR and the natural log of AR assuming no migration ($\Delta {\text{ln}}\left( {{\text{AR}}^{\prime } } \right) = {\text{ln}}({\text{AR}}_{{{\text{current}}}} /{\text{AR}}_{{{\text{zero}} - {\text{migration}}}}$)) for natives

Sex	Hukou type	Primary school or below	Junior high school	Senior high school	College and above
Men	Rural	0.037***	0.019***	− 0.005	− 0.060***
		(0.004)	(0.002)	(0.003)	(0.004)
Men	Urban	− 0.353***	0.007	0.015***	− 0.017***
		(0.016)	(0.005)	(0.004)	(0.003)
Women	Rural	0.017**	− 0.025***	− 0.060***	− 0.112***
		(0.005)	(0.002)	(0.003)	(0.004)
Women	Urban	− 0.170***	− 0.007	0.015**	− 0.024***
		(0.020)	(0.007)	(0.005)	(0.003)

Standard errors in parentheses

* *p* < 0.05, ** *p* < 0.01, and *** *p* < 0.001

The medians of $\Delta {\text{AR}}^{\prime}$ in Fig. 2 and the conditional group means of $\Delta \ln \left( {{\text{AR}}^{\prime}} \right)$ in Table 3 are slightly below zero for native women in most groups, meaning that most of them would have a higher AR if there was no migration, especially for those with rural hukou. The experience for native men varies by education. Native men with primary school or blew education might not feel the difference as they have small ARs in either situation. Those with junior or senior high school education mostly have slightly higher ARs in the current situation, whereas those with a college degree are on average worse off with internal migration. The direction of changes in ARs in Fig. 2 is the opposite of the direction in Fig. 1, and the group means mostly have opposite signs in the corresponding cells in Tables 2 and 3 except for the statistically insignificant ones. The contrast implies that within a certain group by education and hukou type, migrants and natives differ in their experiences in the marriage market during the population redistribution, as Hypothesis 4 suggests. Nonetheless, the $\Delta AR^{\prime}$ s for natives are smaller in magnitude than the ones for migrants, so the externalities are limited.

### Marriage Prospects and Their Predictors

This section shifts the focus from changes in AR due to internal migration to the AR itself. Table 4 shows the estimated group means of the AR controlling for age and current urban/rural residence given the realized geographic distribution of the marriageable population. Comparing ARs for men and women of the same migration status, hukou type, and education level, women have higher ARs than men in all groups except for the ones with senior high school and above education in rural areas. Comparing across education, the AR generally decreases with education for women and increases for men, with a few exceptions in the junior or senior high school education level. Among men without a college degree, those with rural hukou mostly have higher ARs than their peers with urban hukou. The opposite is true for the best-educated men. Comparison between urban and rural hukou shows no consistent pattern for women. There is a clear contrast between migrants and natives. Migrant men mostly have lower ARs than native men with the same hukou type and education, whereas migrant women mostly have higher ARs than their native counterparts.

**Table 4:** Regression estimated conditional group means of AR, controlling for age and current urban/rural residence

	Hukou type	Primary school or below	Junior high school	Senior high school	College and above
Migrant					
Men	Rural	0.511***	0.887***	0.888***	0.857***
		(0.016)	(0.005)	(0.007)	(0.006)
Men	Urban	0.555***	0.575***	0.675***	1.028***
		(0.075)	(0.018)	(0.014)	(0.007)
Women	Rural	1.192***	1.228***	0.957***	0.887***
		(0.025)	(0.006)	(0.008)	(0.007)
Women	Urban	1.308***	1.038***	0.824***	1.089***
		(0.115)	(0.024)	(0.016)	(0.007)
Native					
Men	Rural	0.615***	0.937***	0.908***	0.846***
		(0.006)	(0.003)	(0.004)	(0.005)
Men	Urban	0.513***	0.674***	0.752***	1.055***
		(0.021)	(0.007)	(0.006)	(0.004)
Women	Rural	1.199***	1.118***	0.799***	0.726***
		(0.007)	(0.003)	(0.005)	(0.005)
Women	Urban	1.047***	1.133***	0.934***	1.063***
		(0.026)	(0.009)	(0.007)	(0.004)

Standard errors in parentheses

* *p* < 0.05, ** *p* < 0.01, and *** *p* < 0.001

Table 5 presents the coefficients of the prefectural-level predictors in the regression models about ARs. For men, a higher urbanization rate as measured by the proportion of residents in urban areas is associated with higher ARs. For women, the percentage of tertiary industry in GDP is positively associated with ARs. Women in prefectures with a higher average housing price tend to have higher ARs, though the association is marginally significant. Other prefectural-level variables are not statistically significant predictors.

**Table 5:** Regression of the natural log of AR, coefficients of the predictors

Sex	Men	Women
Education level	Controlled	Controlled
Hukou type	Controlled	Controlled
Age	0.003	− 0.128***
	(0.007)	(0.012)
Age^2^	− 0.005***	0.009***
	(0.000)	(0.001)
Urban residence	0.009	0.021**
	(0.007)	(0.007)
Proportion urban	0.003*	− 0.001
(%)	(0.001)	(0.001)
Proportion with above high school education	− 0.002	− 0.004
(%)	(0.003)	(0.003)
Proportion illiterate	− 0.007	− 0.006
(%)	(0.006)	(0.006)
Disposable income per capita for urban employee	− 0.001	− 0.001
(log, yuan)	(0.003)	(0.003)
Secondary industry in GDP	− 0.002	0.002
(%)	(0.004)	(0.005)
Tertiary industry in GDP	0.008	0.014*
(%)	(0.010)	(0.006)
Number of college student	− 0.061	0.076
(log)	(0.066)	(0.057)
Per capita GDP	0.067	− 0.048
(log, yuan)	(0.113)	(0.119)
Average housing price	− 0.026	0.091 +
(log, yuan/square meter)	(0.050)	(0.053)
Observations	69,312	51,867
*R*^2^	0.361	0.280

Standard errors in parentheses

^+^*p* < 0.1, * *p* < 0.05, ** *p* < 0.01, and *** *p* < 0.001

## Discussion

The empirical analysis largely supports the hypotheses that (1) migrant women mostly improve their marriage prospects via migration, and those of rural origin gain more, (2) migrant men with a college degree are likely to have better marriage prospects after migration, whereas those without the degree lose, and (3) natives’ experiences are different from their migrant peers of the same sex and traits.

The findings reveal a potential conflict between labor market opportunities and marriage prospects in China. For migrants, migrant men substitute for native men in the labor market but might not as bridegrooms due to female status hypergamy (Stark, 1988). This is especially true for migrant men coming from less developed regions and working as blue-collar workers in the receiving place. In the marriage market, they hardly compensate for their relatively low education with other traits such as wealth and family background. These migrant men would have better marriage prospects if they returned to their hometown, so they have stronger incentives than women to return after working for a duration sufficient to accumulate enough wealth for marriage (Liu et al., 2015). In contrast, migrant women with inferior socioeconomic status could find a match and settle down in the receiving place by marrying a native man, leading to a “bride drain” out of sending regions (Fan & Huang, 1998; Mu & Yeung, 2020). For migrant women, improvements in the labor market and the marriage market are more often compatible goals in their migration decision than men, as evident from migrant women’s increased ARs after migration. Another explanation for the observed increase in ARs for migrant women is that women are less likely to sacrifice marriage prospects for job opportunities than men when these two goals are incompatible, resulting in a selective group of migrant women having no such conflict. Such a difference between men and women without a college degree directly results from norms of female status hypergamy, which limits the number of suitable marital partners for migrant men but expands the pool for migrant women. The best-educated migrant men reap the benefits of the redistribution of the marriageable population. A more fundamental cause is inequalities in economic development across regions and urban/rural areas that lead to the concentration of well-paying jobs in big cities, resulting in a mismatch between labor market-driven population distribution and a balancing sex structure needed in the marriage market.

Internal migration also affects the marriage prospects of natives. As Hypothesis 4 suggests and the findings show, natives’ experiences during this population redistribution are different from their migrant peers. Native women mostly face more intense competition in the marriage market, either because suitable partners are moving out from the sending region or competitors are moving into the receiving place. This explains hardships in the marriage market suffered by women in small towns with decent jobs such as civil servants and teachers, as well as the growing number of well-educated single women in their late 20 s or early 30 s in big cities (Fincher, 2014; Jones & Gubhaju, 2009; Magistad, 2013; Ouyang & Ma, 2019; Qian & Qian, 2014). Native men with middle-ranged education enjoy a positive externality of internal migration, which brings in potential brides and takes away competitors. The externalities are small relative to the changes in marriage prospects for migrants, partly because of some countervailing migration flows. For example, for native women in the receiving place, the incoming of migrant men counteracts the increased competition from migrant women. Conceptually, the gains and losses in marriage prospects for migrants and natives would reach an equilibrium where no one wants to change their migration decision given their potential outcomes in the labor market. The equilibrium level of internal migration thus might be responsive to fluctuations both in the economy and in the marriage market (Lee Badgett & Folbre, 2003).

In addition to clear contrasts by sex and migration status, there are also differences by education in terms of gains and losses from internal migration. For men, the best-educated men gain the most among migrants. This is because they are in short supply in the marriage market featured by female status hypergamy and therefore could strategically maximize their marriage prospects. On the other side, the least-educated men have limited options and they hardly improve their marriage prospects through moving to other places. For women, the less educated have more favorable ARs and also tend to gain more as migrants and lose less as natives than the better educated.

The conditional average ARs reveal some structural problems in the marriage market. First, the fact that on average women mostly have higher ARs than their men counterpart reflects a national shortage of marriageable women. The national shortage originates from decades of skewed sex ratios at birth and finds no solution in internal migration (Coale & Banister, 1994; Ebenstein & Sharygin, 2009; Sharygin et al., 2013). Second, the least-educated men face the worst situation in the marriage market with an average AR around 0.6. The intense competition for marriage and the doomed failure for many men in this group have become a risk factor for social stability (Cameron et al., 2019; Huang, 2014; Hudson & Den Boer, 2004). Third, the best-educated urban women have fair marriage opportunities as measured by the AR, so the increasing singlehood among this group is likely to be attributed to changing attitudes toward marriage or a temporary phenomenon due to marriage delay (Yu & Xie, 2021). Fourth, the lack of marriageable men is a real problem for women with senior high school and above education, especially in rural areas. These women do not match with the least-educated men, resulting in a parallel shortage of marriageable men and women. Lastly, native men on average have better marriage prospects than migrant men, indicating a men-specific advantage of local hukou holders over non-local hukou holders. These structural problems might motivate responsive changes in the matching norms, such as greater social acceptance of male status/age hypergamy, especially among groups with the worst marriage prospect under the current situation (Bergstrom & Lam, 1991; Esteve et al., 2012, 2016). The emergence of matrilocal marriage in some developed areas in China might have marked the beginning of such changes (The Economist, 2017, 2021).

This study contributes to the literature on how internal migration affects marriage prospects of migrants and natives. First, it quantifies and compares marriage prospects for unmarried individuals in spatially and socially segregated marriage markets in real and hypothetical settings. The individual-specific ARs under the hypothetical settings best reflect the potential outcomes without migration. Second, this study reveals the impacts of internal migration on the marriage prospects of natives, who do not participate in migration but are affected by the population spatial redistribution. The concept of an externality of migration on natives can be extended to other studies on the impacts of migration. Third, this study demonstrates structural problems within and between the labor market and the marriage market in China, which might have implications for population aging and socioeconomic development in the long run.

### Supplementary Information

Below is the link to the electronic supplementary material.

Supplementary file1 (PDF 2521 kb)

## Data Availability

The datasets and code generated during and/or analyzed during the current study are available from the corresponding author on reasonable request.
