# Supplementary material for: Love is Elsewhere: Internal Migration and Marriage Prospects in China
Source: Eur J Popul. 2023 Mar 2;39(1):6. doi: 10.1007/s10680-023-09658-3 (PMC9981853; doi:10.1007/s10680-023-09658-3)
Supplement: Supplementary file 1 — Supplementary file1 (PDF 2521 kb) [file 10680_2023_9658_MOESM1_ESM.pdf]

Online Resource 1. Weights reflecting local assortative matching patterns in calculating the availability ratio

The weighted version of the Availability Ratio (AR) is the ratio of the weighted number of potentially suitable marital partners for a particular individual to the weighted average number of matches of this individual's matches. The weights capture the level of attractiveness of a potential match and reflect local assortative matching patterns. Two weights are applied in the calculation, one concerning the age gap and the other concerning the education gap between the person and the match. Both weights are province-specific to reflect local prevailing matching preferences. The weights are derived from the empirical distribution of 28,701 marriages formed since 2005 and lasted till 2010 as recorded in the 2010 China census sample data. The final weight is the product of the two weights.

The weight concerning the age gap is a function of the province of location and the age gap between the person and the potential match. The weight is independent of the person's sex and age. The weight for a match with an age gap $\Delta\alpha$ (man–woman) is the proportion of marriage with an age gap $\Delta\alpha$ among marriages with an age gap within [–2, 6]. The sum of the weights equals one. For example, for a woman in Beijing, a man of the same age is counted as 0.197 matches, a man one year older than her as 0.175 matches, and a man one year younger as 0.117 matches. Table A-1 shows the province-specific weights concerning the age gap.

The weight concerning the education gap is a function of the province of location, the person's sex and education, and the potential match's education, being independent of the person's age. The weight for a potential match with education e for a woman with education e' is the scaled proportion of marriage between a husband of education e and a wife of education e' among marriages involving a wife with education e'. Similarly, the weight of a potential match

with education e' for a man with education e is the scaled proportion of marriage between a husband of education e and a wife of education e' among marriages involving a husband with education e. A match with an education gap of greater than one level has zero weight. The sum of the weights equals one. For example, for a woman with senior high school education in Guangdong province, a man with the same education is counted as 0.565 matches, one with a college degree is counted as 0.214 matches, one with junior high school education is counted as 0.220, and one with primary school or less education is counted as 0. Table A-2 shows an example of weights concerning education in Guangdong province. The full set of weights is available upon request.

TABLE A1 Province-specific weights for a match with an age gap $\Delta\alpha$ (man–woman)

	Age gap $\Delta\alpha$ (man–woman)								
Province	–2	–1	0	1	2	3	4	5	6
Beijing	0.053	0.117	0.197	0.175	0.168	0.104	0.086	0.053	0.047
Tianjin	0.071	0.160	0.235	0.214	0.135	0.068	0.082	0.028	0.007
Hebei	0.069	0.132	0.246	0.238	0.147	0.092	0.044	0.020	0.012
Shanxi	0.040	0.081	0.211	0.206	0.170	0.124	0.072	0.058	0.038
Neimenggu	0.075	0.110	0.173	0.203	0.176	0.110	0.066	0.046	0.041
Liaoning	0.050	0.104	0.162	0.196	0.179	0.118	0.089	0.074	0.028
Jilin	0.064	0.097	0.185	0.194	0.168	0.112	0.082	0.067	0.030
Heilongjiang	0.066	0.093	0.186	0.197	0.179	0.100	0.096	0.051	0.033
Shanghai	0.049	0.105	0.178	0.220	0.160	0.101	0.095	0.049	0.042
Jiangsu	0.050	0.126	0.205	0.191	0.175	0.103	0.075	0.047	0.027
Zhejiang	0.045	0.100	0.148	0.184	0.148	0.136	0.118	0.076	0.045
Anhui	0.048	0.110	0.191	0.190	0.169	0.102	0.088	0.065	0.037
Fujian	0.030	0.074	0.162	0.203	0.178	0.133	0.095	0.082	0.044
Jiangxi	0.040	0.082	0.122	0.167	0.207	0.129	0.095	0.094	0.064
Shandong	0.086	0.150	0.223	0.200	0.147	0.093	0.049	0.032	0.020
Henan	0.069	0.125	0.228	0.199	0.163	0.089	0.064	0.037	0.026
Hubei	0.032	0.077	0.140	0.196	0.150	0.139	0.123	0.078	0.065
Hunan	0.032	0.062	0.111	0.182	0.188	0.160	0.131	0.071	0.063
Guangdong	0.036	0.092	0.179	0.166	0.167	0.139	0.111	0.066	0.044
Guangxi	0.044	0.078	0.152	0.170	0.188	0.127	0.112	0.074	0.056
Hainan	0.044	0.081	0.140	0.154	0.184	0.140	0.096	0.088	0.074
Chongqing	0.025	0.076	0.171	0.174	0.154	0.118	0.098	0.096	0.087

Sichuan	0.040	0.085	0.138	0.191	0.159	0.124	0.112	0.077	0.074
Guizhou	0.055	0.087	0.140	0.151	0.177	0.124	0.104	0.091	0.071
Yunnan	0.042	0.109	0.149	0.142	0.142	0.141	0.115	0.076	0.084
Xizang	0.033	0.066	0.189	0.204	0.178	0.124	0.096	0.068	0.042
Shaanxi	0.040	0.095	0.114	0.216	0.169	0.138	0.114	0.078	0.034
Ningxia	0.024	0.071	0.181	0.173	0.220	0.126	0.102	0.087	0.016
**Mean**	0.053	0.117	0.197	0.175	0.168	0.104	0.086	0.053	0.047

Note: author's calculations based on sample data from the 2010 China census.

TABLE A2 An example of weights for a match between a man with education $\boldsymbol{e}$ and a woman with education $\boldsymbol{e'}$ in Guangdong province

Man	Potential match's education e'			
Own education e	Primary and below	Junior high	Senior high	College and above
Primary and below	0.510	0.490	0	0
Junior high	0.062	0.843	0.095	0
Senior high	0	0.399	0.506	0.095
College and above	0	0	0.220	0.780
Woman	Potential match's education e			
Own education e'	Primary and below	Junior high	Senior high	College and above
Primary and below	0.392	0.608	0	0
Junior high	0.036	0.785	0.179	0
Senior high	0	0.220	0.565	0.214
College and above	0	0	0.123	0.877

Note: author's calculations based on sample data from the 2010 China census.

FIG. A1 Boxplots of differences between the AR in the current place of residence and the AR in the hometown (ΔAR) for all migrants by sex, education, and hukou type

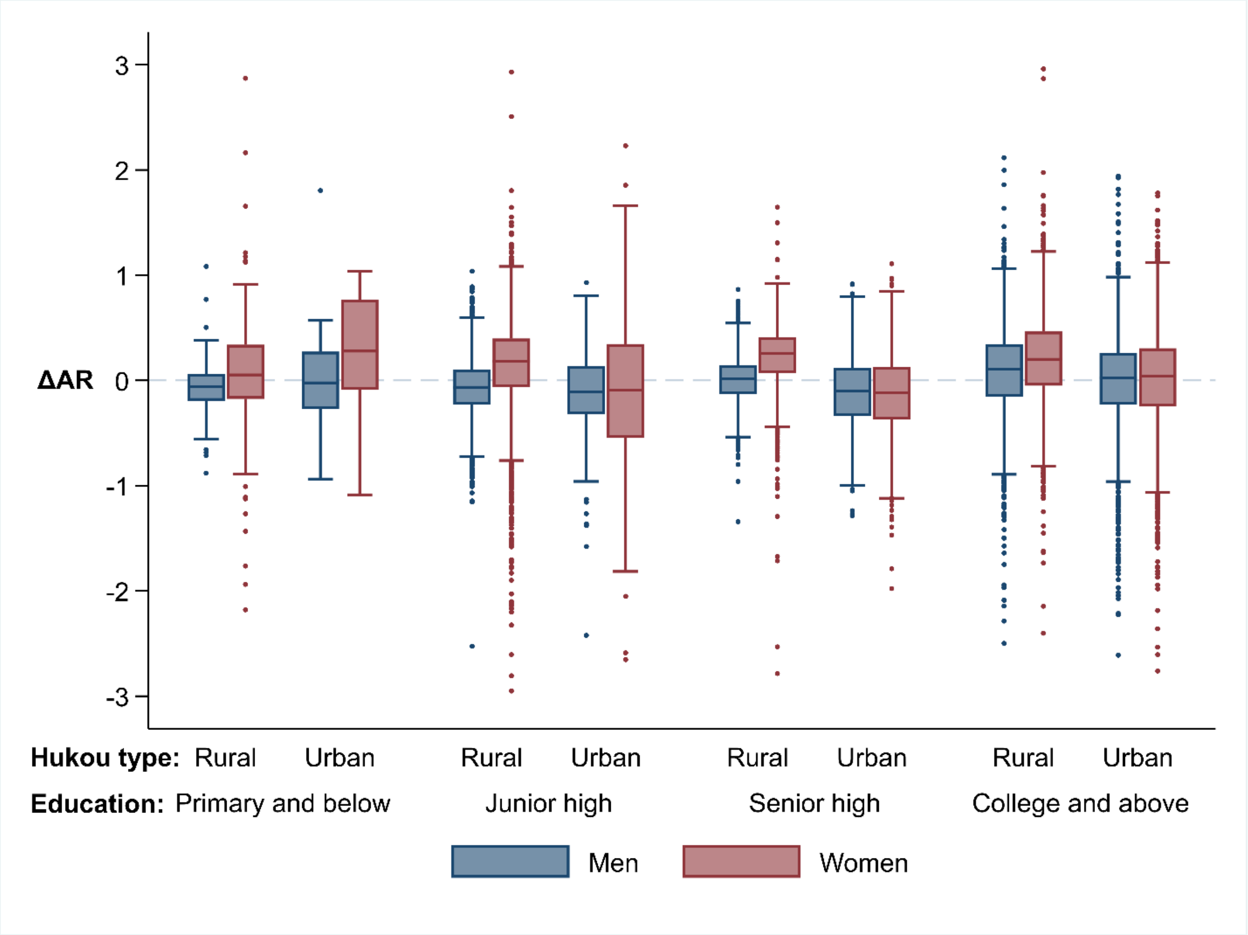

Note: ΔAR = AR in the current place of residence − AR in the hukou address. This graph shows the values for all migrants regardless of migration purpose.

Source: Sample data of 2010 China population census, author's calculations.

FIG. A2 Boxplots of differences between the AR in the current place of residence and the AR in the hometown (ΔAR) for migrants moving for reasons related to the labor market by sex, education, and hukou type, defining the local marriage market at the province level

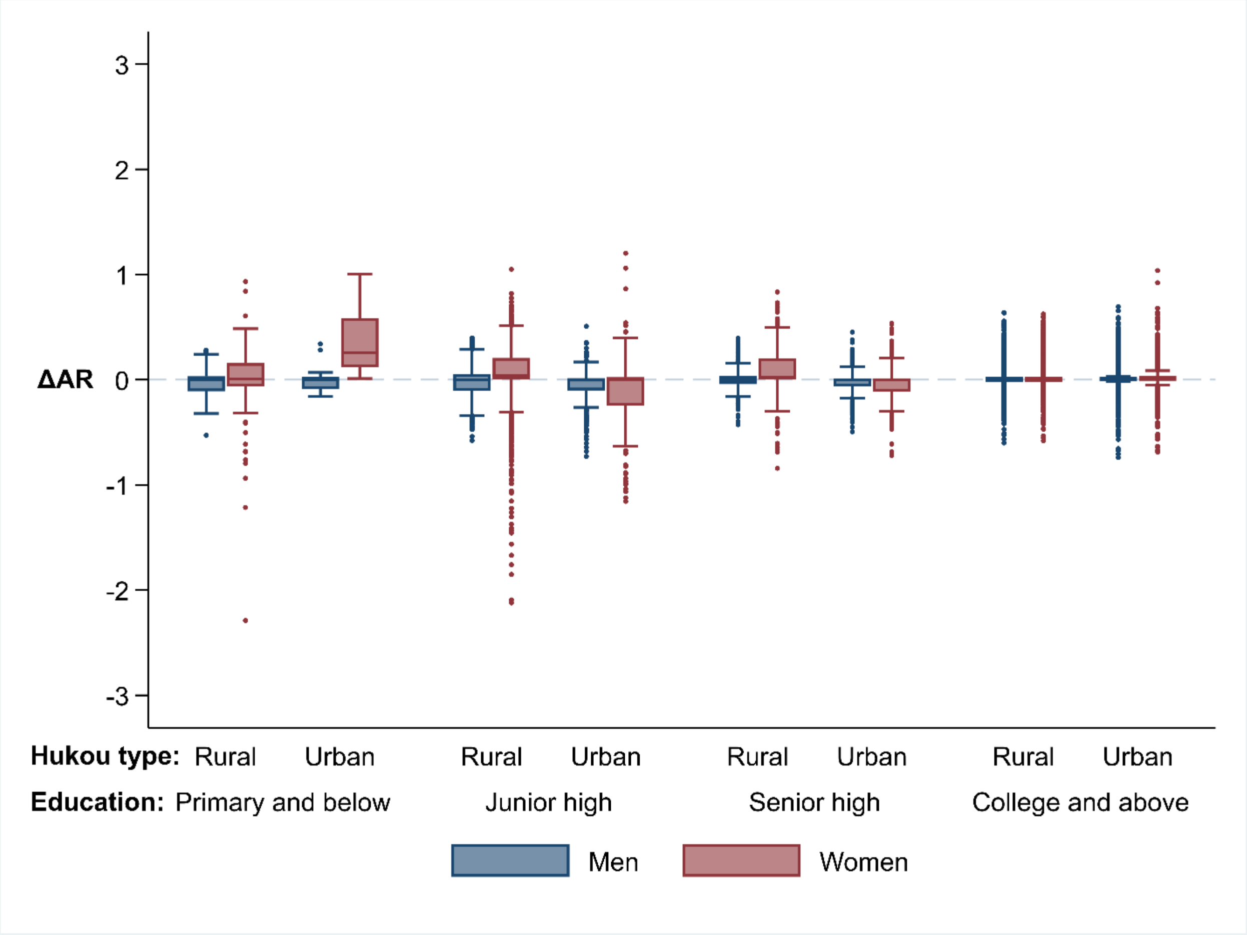

Note: ΔAR = AR in the current place of residence − AR in the hukou address. This graph shows the values for migrants moving for reasons related to the labor market, such as work, business, education, and vocational training.

Source: Sample data of 2010 China population census, author’s calculations.

FIG. A3 Boxplots of differences between the AR in the current place of residence and the AR in the hometown (ΔAR) for migrants moving for reasons related to the labor market by sex, education, and hukou type, allowing for intermarriages between urban and rural hukou

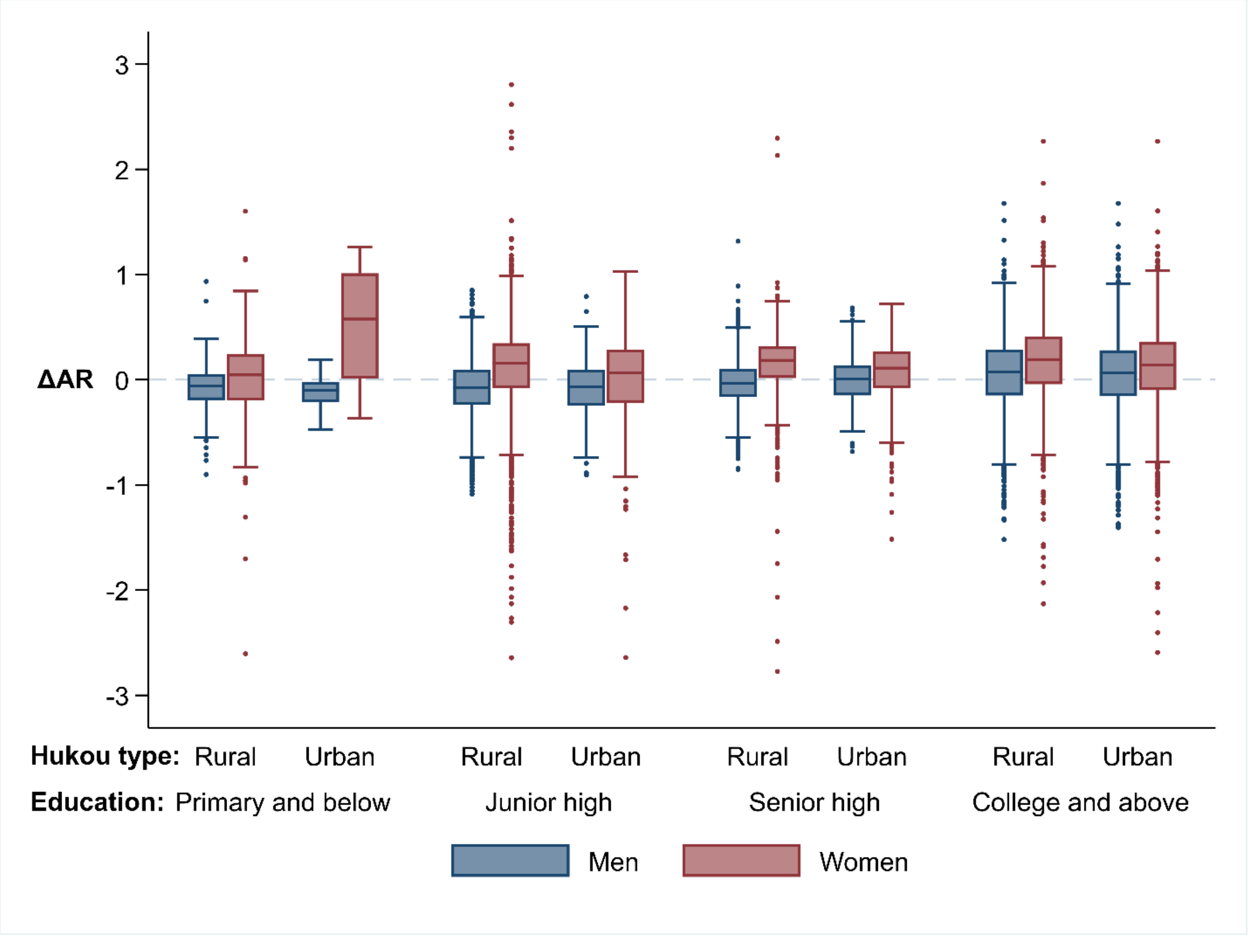

Note: ΔAR = AR in the current place of residence − AR in the hukou address. This graph shows the values for migrants moving for reasons related to the labor market, such as work, business, education, and vocational training.

Source: Sample data of 2010 China population census, author's calculations.

FIG. A4 Boxplots of differences between the AR in the current place of residence and the AR in the hometown (ΔAR) for migrants moving for reasons related to the labor market by sex, education, and current urban/rural residence, assuming local marriage market segregation by current urban/rural residence rather than hukou type

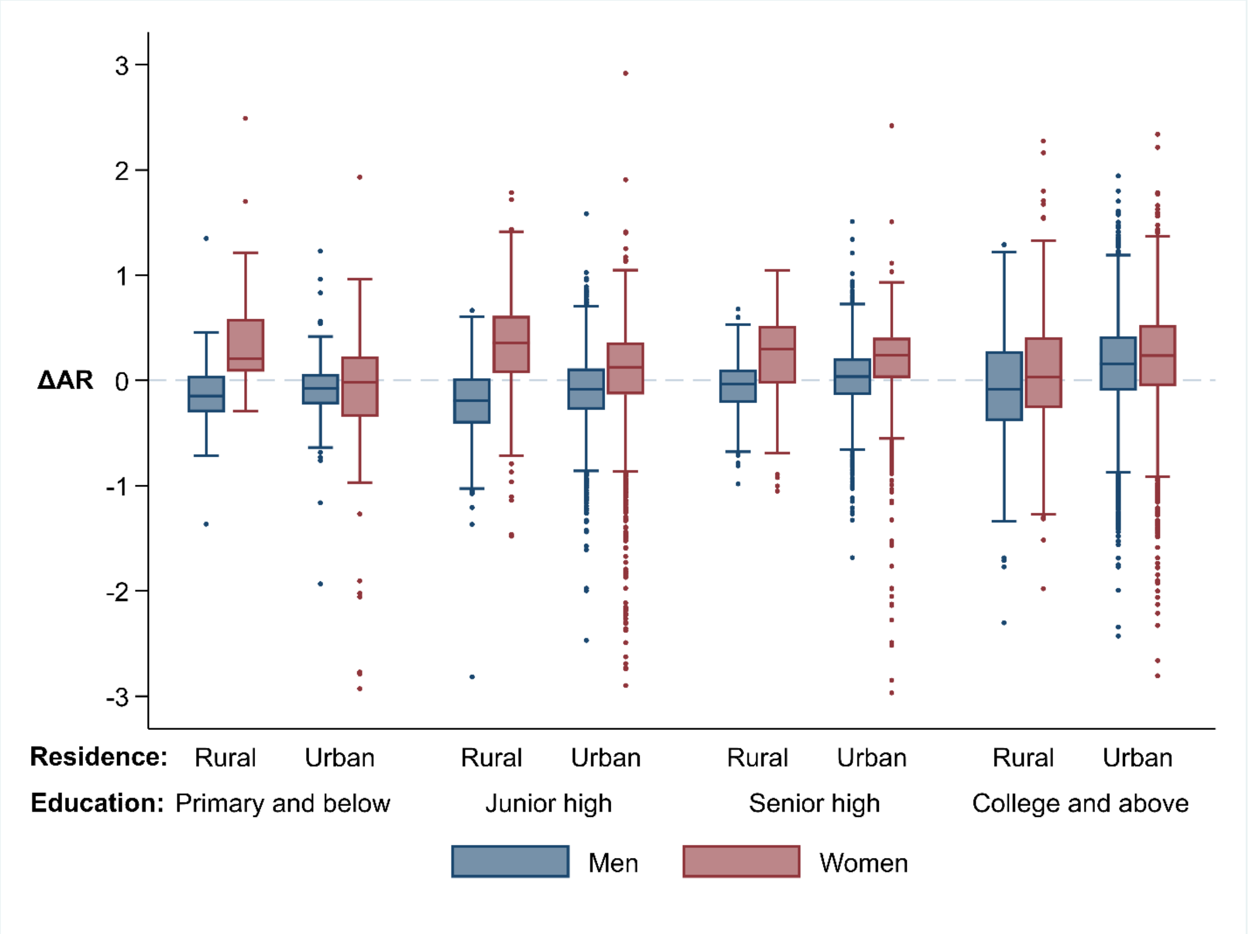

Note: ΔAR = AR in the current place of residence − AR in the hukou address. This graph shows the values for migrants moving for reasons related to the labor market, such as work, business, education, and vocational training.

Source: Sample data of 2010 China population census, author's calculations.

FIG. A5 Boxplots of differences between the current AR and the AR assuming no migration (ΔAR') for all migrants by sex, education, and hukou type

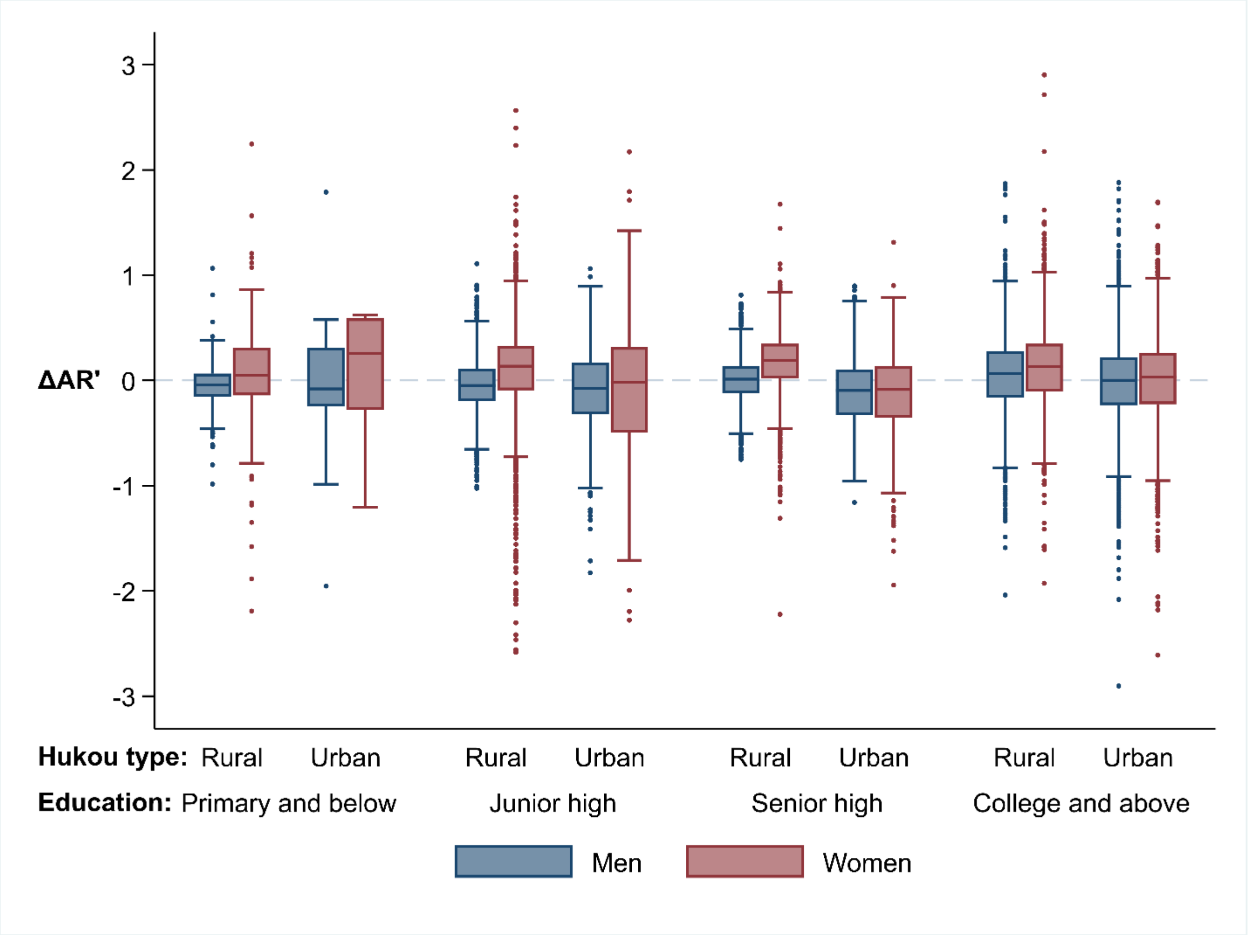

Note: ΔAR = AR in the current place of residence − AR assuming all migrants returned to their hukou address.

Source: Sample data of 2010 China population census, author's calculations.

FIG. A6 Boxplots of differences between the current AR and the AR assuming no migration (ΔAR') for natives by sex, education, and hukou type, defining the local marriage market at the province level

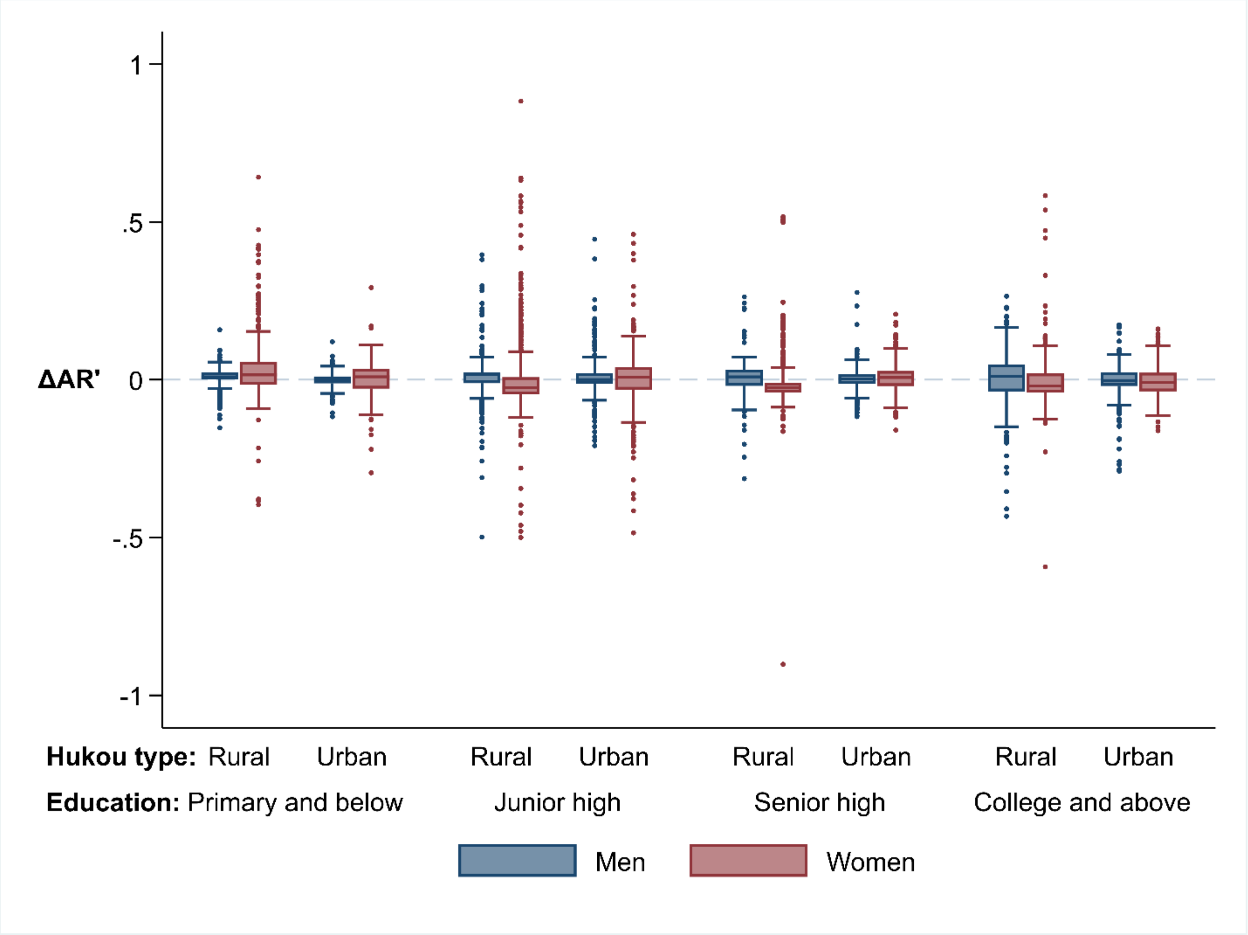

Note: ΔAR = AR in the current place of residence − AR assuming all migrants returned to their hukou address.

Source: Sample data of 2010 China population census, author's calculations.

FIG. A7 Boxplots of differences between the current AR and the AR assuming no migration (ΔAR') for natives by sex, education, and hukou type, allowing for intermarriages between urban and rural hukou

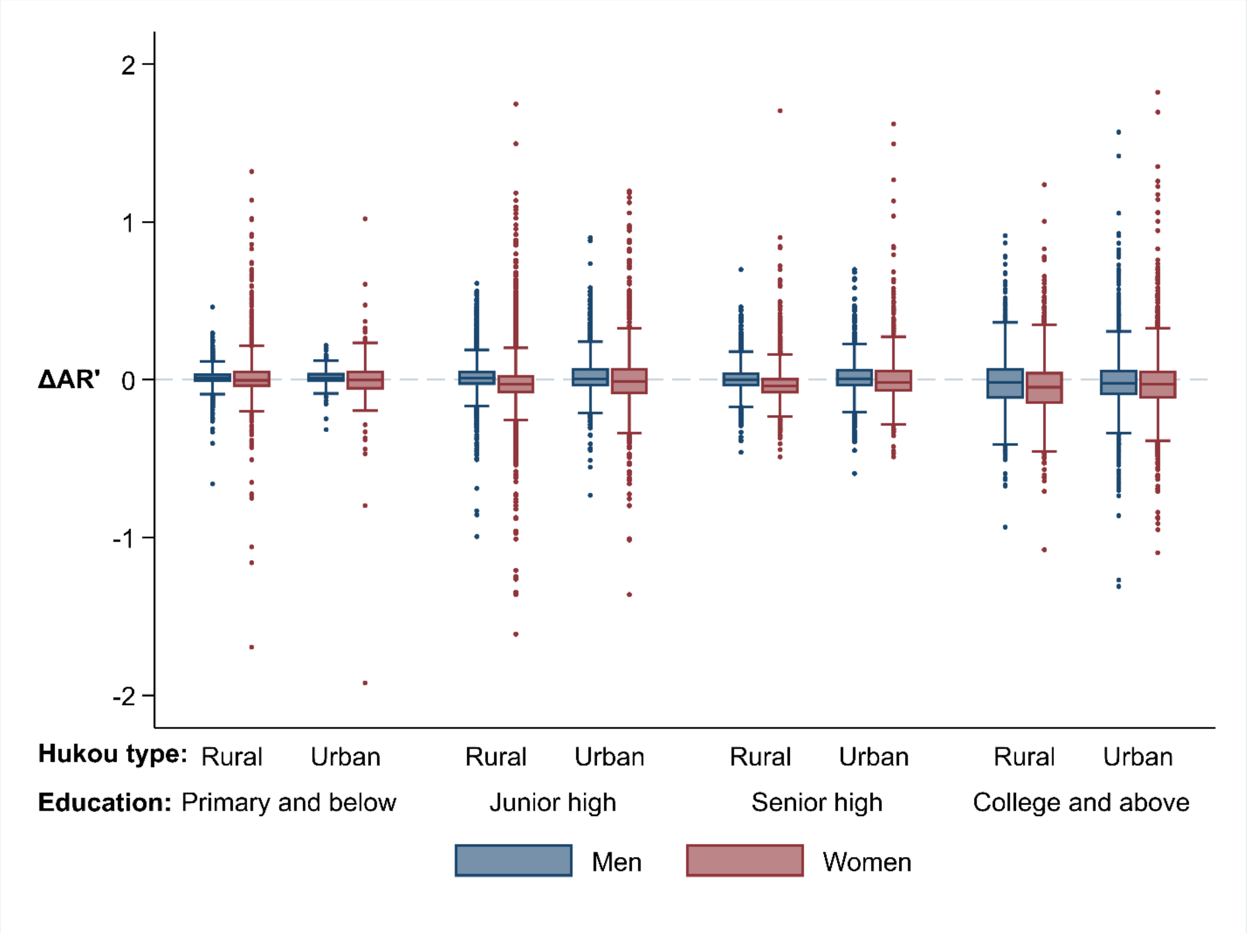

Note: ΔAR = AR in the current place of residence − AR assuming all migrants returned to their hukou address.

Source: Sample data of 2010 China population census, author's calculations.

FIG. A8 Boxplots of differences between the current AR and the AR assuming no migration (ΔAR') for natives by sex, education, and current urban/rural residence, assuming local marriage market segregation by current urban/rural residence rather than hukou type

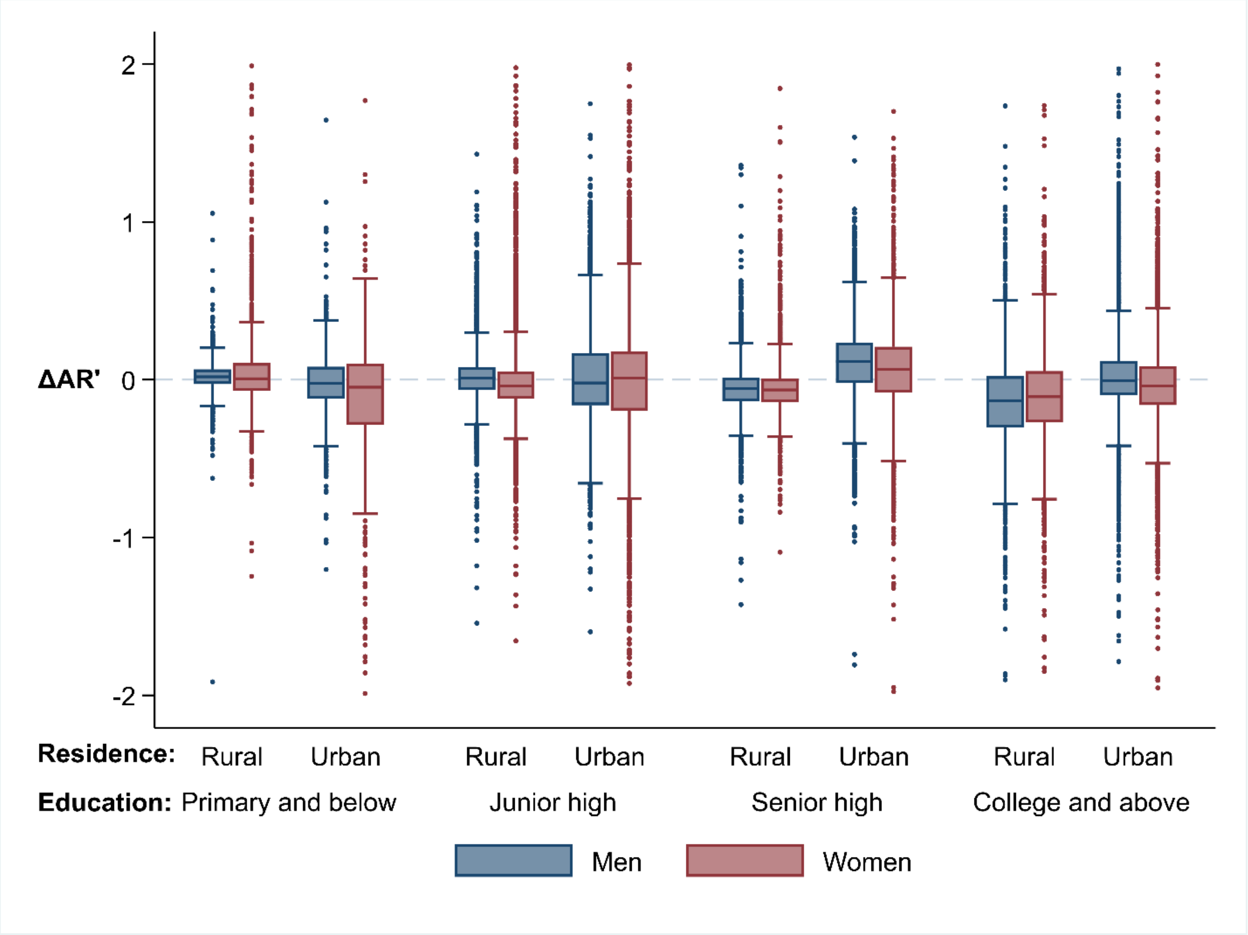

Note: ΔAR = AR in the current place of residence − AR assuming all migrants returned to their hukou address.

Source: Sample data of 2010 China population census, author's calculations.
